# Social visual stimuli increase infants suck response: A preliminary study

**DOI:** 10.1371/journal.pone.0207230

**Published:** 2018-11-09

**Authors:** Emily Zimmerman, Courtney DeSousa

**Affiliations:** Department of Communication Sciences & Disorders, Northeastern University, Boston, MA, United States of America; Chinese Academy of Medical Sciences and Peking Union Medical College, CHINA

## Abstract

This study investigated whether visual stimuli (FACES vs. CARS) combined with the presence of maternal scent can influence suck patterning in healthy infants. Fifteen healthy full-term infants (six months and younger) were exposed to their mother’s scent during a visual preference paradigm consisting of FACES vs. CARS stimuli while sucking on a custom research pacifier. Infants looked significantly longer to the FACES compared to CARS, *p* = .041. Repeated Measures ANOVA revealed a significant main effect for non-nutritive suck (NNS) bursts and visual stimuli (*p* = .001) with the largest differences evident between FACES and when the infant looked away from the visual stimuli (*p* = 0.008) as well as between FACES and CARS (*p* = 0.026). These preliminary findings suggest that infants have more suck attempts when looking at FACES in the presence of maternal scent thereby indicating potent links between visual preference and suck behavior.

## Introduction

Infant suck patterning, or non-nutritive suck (NNS), is a highly patterned motor behavior that emerges soon after birth. NNS occurs at 2 Hz and is organized in bursts of sucking with pause periods for respiration [1]. NNS is controlled by the brainstem suck central pattern generator, or sCPG. The sCPG consists of neuronal networks localized in the brainstem reticular formation that control the activation of motor neurons responsible for new rhythmic motor patterns [2–4]. The sCPG is highly adaptable to descending cortical inputs as well as sensory inputs from the periphery and these signals are capable of modulating the output to lower motor neurons. Previous research has shown that sensory stimulation (e.g., touch, vestibular, olfactory) is capable of altering suck patterning in young infants [2, 5–8].

With vision being the last sense to emerge and develop in infancy, investigation of visual stimulation paradigms and its effect on suck patterning has been lacking. White-Traut and colleagues used eye contact as a form of visual stimulation with preterm infants, as part of a multisensory stimulation paradigm, and found that infants who received the multisensory stimulation had enhanced maturation of suck development [9]. Marshall and colleagues used high contrast visual patterns taped on the side of preterm infants’ incubators during their hospital stay and showed that these infants had fewer state changes and stronger visual skills compared to the control group [10]. Taken together, these research findings suggest that visual sensory stimulation may promote behavioral organization and suck pattern development in preterm infants. However, it remains unclear how different types of visual stimuli will alter these responses.

Durand and colleagues examined infants’ attention response to visual stimuli that was social and non-social [11]. A unique aspect of this study was that the investigators examined the extent to which the presence of maternal odor can affect the infants’ attention response. Visual stimuli were composed of a female face (social stimuli) presented side-by-side with a car (non-social stimuli) on a white-background screen. Placement of the paired stimuli was counterbalanced throughout the four (30s) trials of the study between the experimental and control groups. Infants in the experimental group were exposed to their mother’s body odors presented on a t-shirt, which was worn by their mother for three consecutive nights prior to the study. Infants’ visual exploration was recorded with an eye-movement tracking system. They found that infants looked significantly longer at the social faces than the non-social cars, and this finding was more robust when infants were simultaneously introduced to their mother’s scent as opposed to the control group without exposure to their mother’s scent. Interestingly, Doucet, Soussignan [12] examined the responses of infants facing their mother’s breast in a scentless (covered breast) versus four scent conditions: full exposed breast, only nipple exposed, areola only exposed and milk exposed. These data showed that infants were more orally active (defined as rooting, licking, sucking, and chewing) when facing the breast during any of the odorous breast conditions compared to the scentless condition. The scentless condition also resulted in more crying with less eye opening. While these data revealed the potent influence of maternal scent on oral movements, the study did not quantitatively examine NNS with a pacifier nor the effect of social visual stimuli. Given the previous research showing the influence of maternal olfactory cues and feeding behaviors [8, 13], a logical next step to the Durand study was to include suck dynamics to examine its relation to visual preferences. Therefore, the goal of this study was to determine if and to what extent visual stimuli (FACES vs. CARS) combined with the presence of maternal scent can influence suck patterning in healthy infants. It was hypothesized that infants would suck with higher suck amplitudes, more suck cycles/bursts, and more suck bursts when looking at the FACES compared to the CARS.

## Materials and methods

### Participants and ethical statements

The protocol was approved by the Institutional Review Board at Northeastern University (Protocol Number: 16-04-06) and parental consent was attained prior to the start of the study. Mother-infant dyads were recruited through the Lab Facebook page, local websites that mothers frequent (e.g., babysitting/parenting forums, blogs, and discussion boards) and flyers posted across the local community. Inclusion criteria included healthy full-term infants six months and younger. Exclusion criteria for caregivers include caregivers under the age of eighteen and non-English speaking persons. Exclusion criteria for infants include preterm birth, serious medical diagnoses, hearing difficulties, vision difficulties, sensitive skin, allergies to adhesive, and/or a history of diagnosed medical conditions or feeding complications. Parking was paid for and parents were compensated with a small infant toy for their participation.

Eighteen full-term infants completed this study. However, eye preference, NNS, and cardiorespiratory data were collected but not analyzed on three infants due to behaviors (e.g., crying) resulting in no data. Therefore, 15 subjects (birth weight range 6 pounds 10 ounces to 9 pounds 2 ounces), healthy infants (10 male/ 5 female), six months and younger (average age at testing mean 4.75 months (± 1.07), range: 3 month 6 days to 6 months 25 days), with no reported medical complications, completed this study. Majority of the infants were breastfed (*n* = 13) and 11 infants had prior pacifier history, with 9 infants still actively using a pacifier at the time of the study. For subject 13, only cardiorespiratory and eye preference data was attained due to technical difficulties with our NNS device.

### Stimuli (olfactory and visual)

#### Visual stimuli

**Fig 1:**
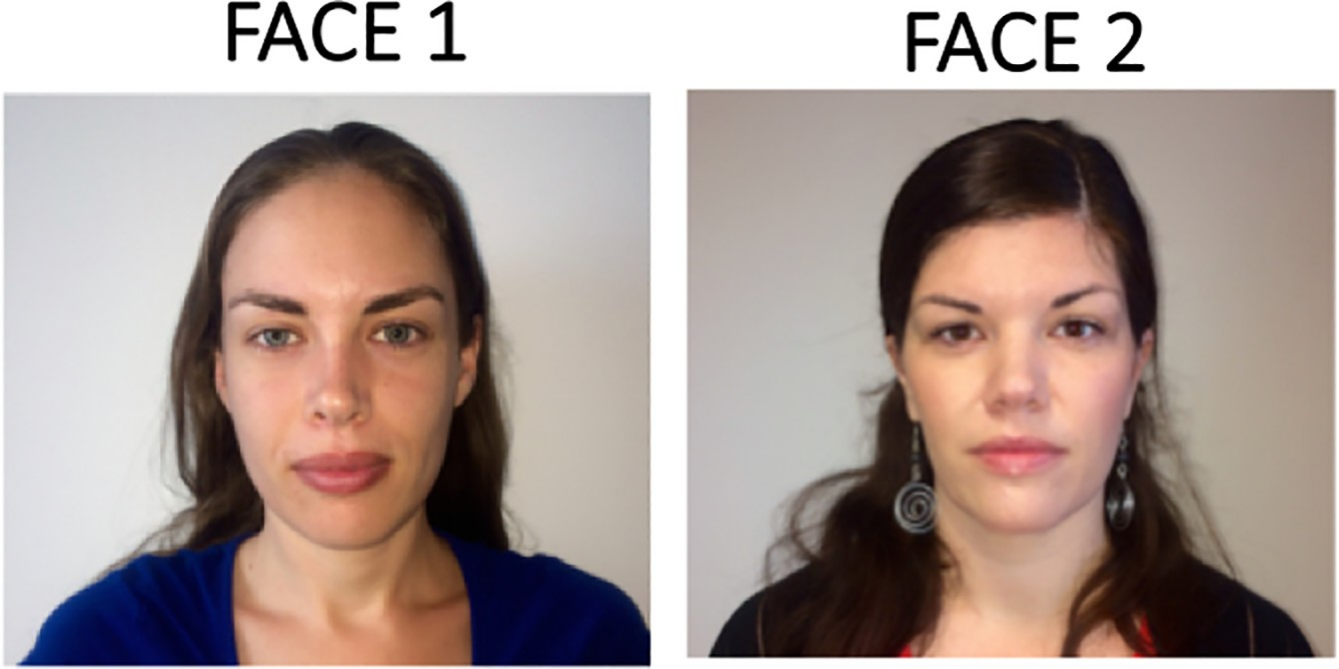
FACE visual stimuli. The FACES (woman 1 and woman 2) that were presented during the study. The subjects in the photograph have given written informed consent, outlined in the PLOS consent form, to publish their pictures.

The visual stimuli consisted of FACES and CARS. The two female FACES used in the study were photographed facing forward with a neutral expression and with their gaze forward. These females had similar expressions and hair color, see Fig 1. The two CAR stimuli were of two cars, one red sedan and one white SUV. These CARS were parked facing forward on a left slant so the infant could visual the front and full right side of the CAR. These stimuli were chosen purposefully to be similar to the images used in the study by Durand and colleagues.

The presentation of the visual stimuli (FACES and CARS) consisted of four (30s) trials. Two pair sets containing a FACE and a CAR presented side-by-side on a computer screen, twice consecutively with a 1s inter-trial period, and counterbalanced for side of presentation across two trials. Each set of stimuli consisted of a different female FACE and a different CAR.

#### Olfactory stimuli

Approximately one week preceding the participant's visit to the lab, the consent form and t-shirt were sent (enclosed in a paper bag, and enclosed in a zip-locked hermetic plastic bag) to the participant’s address. Following the methods of Durand and colleagues, the mothers were instructed to wear the t-shirt (on skin) for three consecutive nights preceding their visit to the lab. The mothers were instructed to place the t-shirt in the bags (as they received it) when they were not wearing them. Maternal t-shirts were discarded after the study.

#### Experimental setting and procedures

Mothers were instructed to bring their infant to the lab approximately one hour prior to their scheduled feeding time. Upon arrival for the study, infants were fitted with the respiratory and heart-rate sensors, which served as control variables and are part of our ongoing data collection protocol to ensure that cardiorespiratory patterning remained stable across conditions. The study took place in the Infant Discovery Room within the lab, which is a sound treated room with a fabric wrapped acoustic panel on one wall designed to reduce noise. Throughout the study, the infant was placed in a baby seat in a semi-inclined position directed toward the computer screen. The screen was approximately 24 inches from the infant. There were three researchers in the room during the testing. One researcher was behind the video camera, which was behind and slightly above the computer screen, to ensure that it was working properly and had a clear view of the infant’s head. Once positioned, this researcher did not move and stayed behind the screen observing the study. The other researcher was holding the custom pacifier for the infant. This researcher would offer the infant the custom pacifier by gently stroking the infant’s cheek and then would crouch behind the infant’s chair so that she was not visualized by the infant during the study. The last researcher was in control of the data acquisition system to ensure that the physiological data was being collected accurately. Researchers were instructed to not wear any perfume or any personal care products with a scent and to not engage or look at the infant during the testing. The caregivers observed the study from our observation room, which is equipped with a one-way mirror looking into the discovery room. The caregivers were instructed not to come into the room unless the researcher said it was okay to come comfort the baby if he/she was crying. The lights were dimmed for the entire experiment.

Given the findings from Durand and colleagues showing that infant look duration was even more robust when looking at social faces in the presence of olfactory stimulation[11], all infants were presented with their mother’s t-shirt, folded and fixated on the infant’s upper chest, right below the chin. As the stimuli were presented, the infant was offered a Soothie^TM^ pacifier attached to our custom NNS device, which was connected to a pressure transducer to measure the infant’s suck patterning. Each pacifier was only used one time per subject and was discarded after every use. While the infants looked at the computer screen their eye movements were captured with a video camera.

### Dependent variables (recording equipment)

#### Cardiorespiratory variables

A Pneumotrace respiratory belt transducer (ADInstruments, Bella Vista, Australia) was placed around the infant’s abdomen to measure respiratory rate (RR), and Softrace small radiotranslucent (RTL) electrodes were placed on the infant’s chest to measure cardiac data (heart rate [HR] and heart rate variability [HRV]). The cardiac signals were amplified using a BioAmp (ADInstruments, Bella Vista, Australia). The sampling rate for both RR and HR was set for 1000/sec. The RR range was set for 200 mV and the HR/HRV range for 5 mV. RR, HR and HRV data were filtered by a Mains filter to reduce electrical noise. A high pass filter of 0.3 Hz was applied to the HR data during data collection on all participants to reduce movement artifact. High frequency versus low frequency HRV was determined by the power spectra energy pre-set by LabChart Pro Software (ADInstruments, Bella Vista, Australia) using the HRV 2.0 Add-On with the cut-off of 0.15–0.45 hertz (Hz) for high frequency HRV and 0–0.15 Hz for low frequency HRV.

#### NNS variables

The NNS system utilized a pressure transducer (Honeywell TruStability HSC Series Pressure Sensor) that was housed outside of the pacifier handle in black box next to the data acquisition system. The pressure transducer was calibrated before each participant. Data acquisition was completed using the ADInstruments Power- Lab (16/35) system, and LabChart Pro software was used to analyze cardiac, respiratory, and NNS dynamics. Suck dynamics included NNS burst duration (seconds), cycles per burst, cycles per minute, bursts per minute, frequency (hertz), and amplitude (centimeters of water). All data were saved as the participant’s ID number in an effort to avoid researcher bias during data analysis.

#### Look duration

A Samsung camcorder collected data on infant look duration for each of the stimuli displayed on the computer screen. After the study was completed, duration of infant looking was measured with a custom-written program that recorded the amount of time infants looked at each stimulus.

#### Reliability

The reliability (inter and intra) of eye tracking measures was evaluated by re-measuring the eye preference data chosen at random. Inter-Rater reliability for 21% of the data was high for looking toward stimulus duration (r = .945), looking away from stimulus duration (r = .917), red CAR look duration (r = .999), white CAR look duration (r = .974), woman FACE 1 look duration (r = .993), and woman FACE 2 look duration (r = .994). Intra-Rater reliability for 42% of the data was high for looking at stimulus duration (r = .996), looking away from stimulus duration (r = .997), red CAR look duration (r = .971), white CAR look duration (r = .962), woman FACE 1 look duration (r = .979), and woman FACE 2 look duration (r = .993).

#### Statistics

For look duration data, a test of normal distribution was completed for all of the dependent variables using the Shapiro-Wilk Test, which revealed that all of the dependent variables were normally distributed except for the white CAR data. Therefore, a non-parametric Chi-Squared test was used for that the white CAR vs. red CAR comparison. To determine the relation between sucking, cardiorespiratory and visual preference data, separate Repeated Measures ANOVA (RM ANOVA) were completed. RM ANOVAs were conducted for each NNS (burst duration, cycles per burst, cycles amount, bursts amount, frequency, and amplitude) and cardiorespiratory (respiratory rate, heart rate, low frequency HRV, high frequency HRV and low-to-high HRV ratio) dependent variable, with condition (FACES, CARS, Look Away from Stimuli) repeated within participants. All post-hoc comparisons were Bonferroni corrected to reduce the risk of Type I error. Bivariate correlations were examined for NNS Bursts, NNS cycles, and eye gaze differences across trials.

## Results

**Fig 2:**
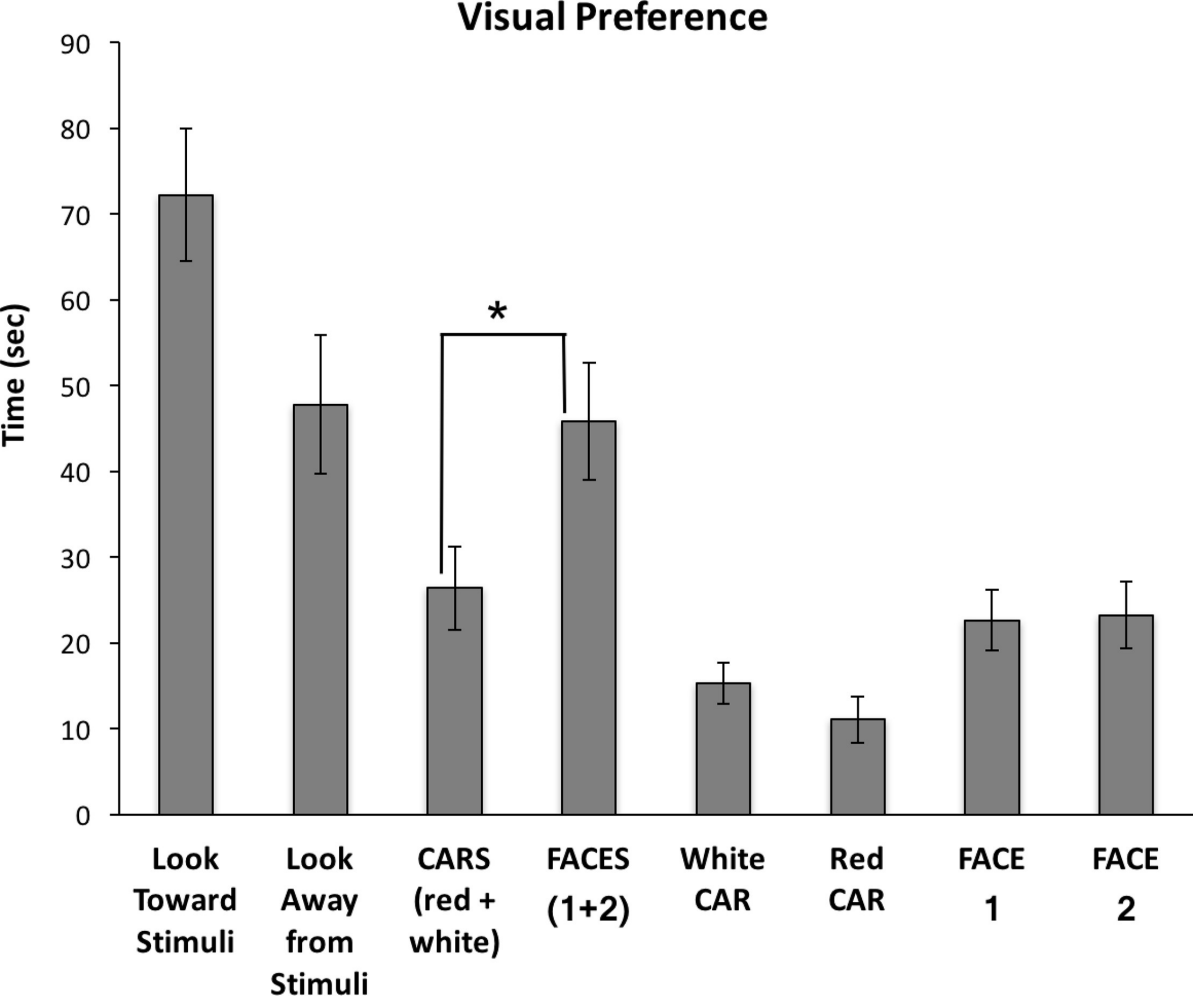
Look Duration and Visual Stimuli. Look duration (seconds) is compared for the time looking toward the various stimuli and looking away from the stimuli for the 4 (30s) trials.

Infants looked significantly longer at the FACES (M = 45.84, SD = 26.48) than the CARS (M = 26.36, SD = 18.67); t(14) = -2.24, *p* = .041, see Fig 2. When looking at the CARS, there was no significant difference in the look duration for the red (M = 15.97, SD = 10.39) compared to the white CAR (M = 11.75, SD = 9.28); χ^2^ (1) = 78.46, p = 1.00. There were no significant differences in the look duration between the FACES (Woman 1 M = 23.22, SD = 15.34; Woman 2 M = 22.62, SD = 13.69); t(14) = -.191, *p* = .851. Lastly, there were no significant differences between the look duration toward the stimuli (M = 72.20, SD = 30.08) versus looking away from the stimuli (M = 47.79, SD = 31.21); t(14) = 1.582, *p* = .136.

**Fig 3:**
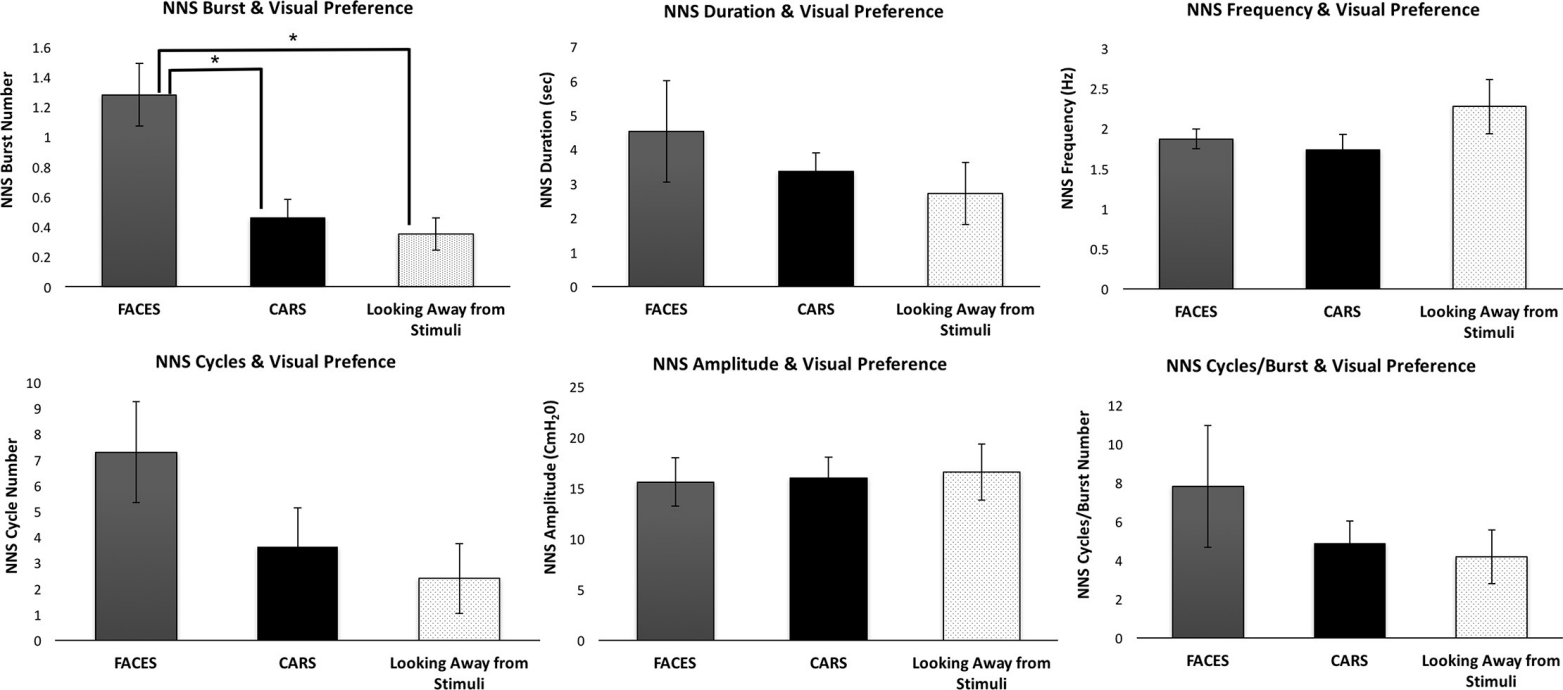
Non-nutritive suck and visual preference. The non-nutritive suck variables are compared during FACES (gray), CARS (black) and looking away from stimuli (dotted bar) across the four (30s) trials. Error bars represent the standard errors of the mean (SEM). *: p<0.05.

Separate Repeated Measures ANOVA were completed on the NNS and visual preference data, see Fig 3. There was a significant main effect for NNS bursts and visual stimuli (F(2, 26) = 8.975 *p* = .001, ηp^2^ = .408). Pairwise comparisons, with a Bonferonni post-hoc adjustment, revealed a significant difference between the FACES versus the looking away from stimuli conditions (*p = 0*.*008*, 95% CI [.240, 1.628]) and between FACES and CARS (*p = 0*.*026*, 95% CI [.090, 1.553]). NNS Cycles/min did approach significance (F(2, 26) = 2.905 p = .073, ηp^2^ = .183) with the largest difference evident between FACES (M = 7.29, SD, 7.14) vs. Look Away (M = 2.39, SD = 472) but this did not reach significance. Additionally, there were no significant differences between NNS burst duration (F(2, 10) = .743 p = .500, ηp^2^ = .129), NNS amplitude (F(2, 10) = .133 p = .877, ηp^2^ = .026), NNS Frequency (F(2, 10) = 1.899 p = .200 ηp^2^ = .275, and NNS Cycles/Burst (F(2, 10) = .994, p = .371, ηp^2^ = .166) across conditions.

The relation between NNS bursts and eye gaze duration while looking at the FACES across trials was explored in more detail. To do this, the differences in eye gaze duration (e.g., eye gaze duration in Trial 4 minus the eye gaze duration in Trial 1) and NNS bursts (e.g., NNS burst number in Trial 4 minus the NNS burst number in Trial 1) was calculated comparing trials (4 vs. 1, 3 vs. 1 and 2 vs. 1), which resulted in two new continuous difference variables. Bivariate correlations using these variables showed that the eye gaze duration and NNS burst differences were significantly correlated for Trial 4 vs. 1 for FACES, see Table 1. However, there were no significant correlations for the differences between Trial 3 vs. 1 nor for Trial 2 vs. 1 for FACES. For comparison, this same process was completed for NNS burst and eye gaze duration while looking at CARS and LOOK AWAY revealing no significant correlations.

**Table 1:** Bivariate correlations between NNS burst and eye gaze differences across trials.

	Trial 4 vs. 1	Trial 3 vs. 1	Trial 2 vs. 1
FACES	.562*	.440	.202
CARS	.086	.294	.018
LOOK AWAY	.231	.010	.221

Note

*p < .05

**Fig 4:**
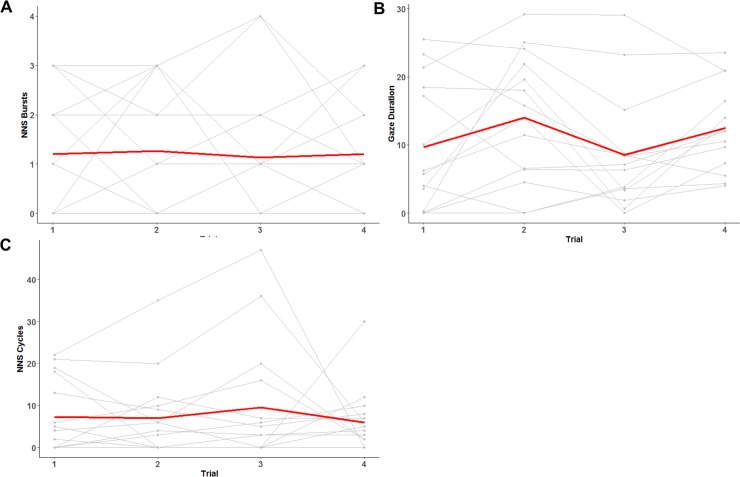
**Spaghetti plots for NNS Bursts (A), Gaze Duration (B), and NNS cycles (C) across trials.** Each infants’ data is in light gray and the average is shown in dark red.

Individual data for a subset of the dependent variables, NNS bursts, NNS cycles and eye gaze duration during the FACES condition, were plotted to examine individual differences, see Fig 4. These spaghetti plots reveal a vast amount of variability across NNS bursts, NNS Cycles, and eye gaze duration.

Separate Repeated Measures ANOVA were completed on the cardiorespiratory and visual preference control data. There were no significant differences between respiratory rate (F(2, 26) = .9128 p = .871, ηp^2^ = .011), heart rate F(2, 26) = 2.384 p = .142, ηp^2^ = .155), low frequency HRV (F(2, 26) = 2.26 p = .124, ηp^2^ = .148), high frequency HRV (F(2, 26) = 1.26 p = .299, ηp^2^ = .089) and the low-to-high frequency ratio (F(2, 26) = .307 p = .738, ηp^2^ = .023) across conditions.

## Discussion

This preliminary study aimed to determine if and to what extent visual stimuli (FACES vs. CARS) combined with the presence of maternal scent influences suck patterning in healthy infants. Durand and colleagues showed that infants looked significantly longer at the FACE vs. CAR without the mother’s scent, and this difference was even more pronounced in the presence of maternal scent. Because of this finding, the current study only used the maternal scent condition and found that infants looked significantly longer at the FACES compared to the CARS. This finding is also consistent with the previous research showing that infants’ have a heightened responsiveness to the human face [14, 15] with a particular preference for faces over objects [16, 17]. There were no other significant differences between the visual preferences in this study.

We expanded on the Durand study to show that infants increase their suck response when looking at FACES compared to CARS. The one NNS dependent variable that was significant, NNS bursts, indicates that the infant is attempting to suck more often when they are looking at FACES. It could be argued that the infant was indeed sucking more during FACES because the infant looked longer at the FACES compared to the other conditions and therefore had more of an opportunity to suck. However, on average, infants spent a longer duration of time looking away from the stimuli (1.71 seconds longer) compared to FACES and yet did not yield more NNS bursts during that time. Additionally, the analysis that was completed to examine the change in NNS burst and eye gaze duration across trials confirmed the finding that as eye gaze increased while looking at the FACES, so did NNS burst amount. These associations were less robust while looking at the CAR and LOOK AWAY conditions. These trends need to be examined in more detail with a larger sample size.

Although there were compelling group differences in these data, NNS is very complex and must be considered on an individualized basis. Previous NNS data using the same custom NNS pacifier device has shown tremendous individual NNS variation throughout the first 6 months of life [18]. Examining each participant’s data on the NNS burst, NNS cycles, and eye gaze duration further confirmed the large amount of variability present across subjects and trials. This further supports the need for future studies with a larger number of subjects and with a narrower age window in an effort to reduce the variability.

The results from this study have several implications as to the role of the suck patterning in relation to early preferences as well as the connectivity of suck and visual systems. High amplitude sucking has been widely used in psychological studies as an indicator of discrimination or preference. Previous research has been particularly robust in regards to infant speech perception, such as early speech discrimination [19], phonetic discrimination [20], and perceived word stress [21]. More recently, these paradigms have shifted to focus on helping infants in the neonatal intensive care unit (NICU) learn to suck. For example, a pacifier-activated-music-player was used with premature infants to show that when an infant’s suck reached a pre-set amplitude threshold, the infant was rewarded with a lullaby song of their mother singing, which in turn significantly increased their oral feeding skills [22]. Another study by Butler and colleagues found that the degree of maternal pitch modulation predicted an increase in infant suck bursts[23]. These data combined with others findings from our lab [7, 18] have shown that more suck variables beyond infant amplitude can indicate early infant preferences and should be utilized both in clinical and research settings.

While Doucet and colleagues (2007) showed that infants were more orally active when facing the breast compared to the scentless condition, this study is the first to quantitatively examine NNS response during maternal scent and show that when infants look at FACES, they produced more NNS suck bursts. The exact pathways by which this interaction occurs remains unknown, but we speculate that in addition to the aforementioned sense, vision is another sense that is capable of modulating suck activity through the sCPG circuitry, which is extremely adaptable to sensory inputs. Another possible neurological link between suck and visual preferences is mediated through infant arousal. It is well documented in the literature that NNS increases infant attention and improves behavioral responsiveness [24–27] and increased attention enhances visual preferences [28]. More in-depth data on attention is needed to explore this phenomenon in more detail.

The finding that infants have more suck bursts when looking at FACES has significant clinical implications. If looking at FACES increases infant suck bursts, this is a very simple and cost-effective way to enhance suck in infants who exhibit poor sucking skills. In fact, these types of sensory (olfactory, orocutaneous, vision) cues presented to the infant are often easier to perceive when they are presented multimodally than when they are presented one sense at a time [29]. This is because multimodal stimulation plays a key role in organizing early selective attention and in turn in directing early perception, learning, and memory [29–32]. Thus, FACES paired with NNS and/or bottle feedings in the presence of maternal scent would likely have robust effects on clinical outcomes in the NICU, such as sucking development, behavioral state control, and oral feeding.

Cardiorespiratory control variables were not significantly different across stimuli indicating that infants were able to modulate their suck patterning and visual preferences while maintaining stable and consistent cardiorespiratory patterning, which was expected for this full-term healthy infant cohort. Other studies have examined cardiorespiratory patterning in relation to heart-rate changes as a function of the stimulus onset [28, 33, 34] and measured the proportion of time spent in the various attention phases. As mentioned earlier, the cardiorespiratory signals collected in the current study were part of an ongoing protocol in the lab, so these variables were only examined as averages during the visual preference conditions and were not parsed based on time epoch surrounding the initial stimulus onset. However, future studies should examine this in more detail to further determine the role of attention in mediating this visual and suck response.

Despite the contributions of this study, a number of potential limitations should be acknowledged. Infants in this study were age six months and younger and some had difficulty separating from their mothers for the duration of the study, resulting in infants crying, which affected the data for a few participants. The age range for this study was large and future studies should be focus on a narrower age group. Subsequent studies should consist of a larger sample size and more conditions. For example, just maternal scent while sucking, just visual stimuli while sucking, and then scent plus visual stimuli while sucking, to parse out the role of maternal scent in the outcome. Expanding on the social stimuli to include the infant’s own mother or father or even an image of the participant, would be another logical next step. Future studies should also inquire about the weaning status and prandial state of the infants in the study.

## Conclusions

Preliminary findings show that in the presence of maternal scent, infants look significantly longer at FACES compared to CARS. Additionally, infants have significantly more suck bursts when looking at FACES compared to CARS or when they are looking away from the stimuli. These findings indicate potent links between suck behavior, maternal scent and visual preference that should be explored in more detail.
